# Using Creative Problem Solving (TRIZ) in Improving the Quality of Hospital Services

**DOI:** 10.5539/gjhs.v7n1p88

**Published:** 2014-08-15

**Authors:** Behrouz LariSemnani, Rafat Mohebbi Far, Elham Shalipoor, Mohammad Mohseni

**Affiliations:** 1 Department of Business Administration, Payame Noor University, Tehran, Iran; 2 Department of Health Management, Qazvin University of Medical Sciences, Qazvin, Iran; 3 Payame Noor University, Karaj, Iran; 4 Research Center for Health Services Management, Institute for Futures Studies in Health, Kerman University of Medical Sciences, Kerman, Iran

**Keywords:** TRIZ, hospital, quality, SERVQUAL

## Abstract

TRIZ is an initiative and SERVQUAL is a structured methodology for quality improvement. Using these tools, inventive problem solving can be applied for quality improvement, and the highest quality can be reached using creative quality improvement methodology. The present study seeks to determine the priority of quality aspects of services provided for patients in the hospital as well as how TRIZ can help in improving the quality of those services. This Study is an applied research which used a dynamic qualitative descriptive survey method during year 2011. Statistical population includes every patient who visited in one of the University Hospitals from March 2011. There existed a big gap between patients’ expectations from what seemingly is seen (the design of the hospital) and timely provision of services with their perceptions. Also, quality aspects of services were prioritized as follows: keeping the appearance of hospital (the design), accountability, assurance, credibility and having empathy. Thus, the only thing which mattered most for all staff and managers of studied hospital was the appearance of hospital as well as its staff look. This can grasp a high percentage of patients’ satisfaction. By referring to contradiction matrix, the most important principles of TRIZ model were related to tangible factors including principles No. 13 (discarding and recovering), 25 (self-service), 35 (parameter changes), and 2 (taking out). Furthermore, in addition to these four principles, principle No. 24 (intermediary) was repeated most among the others. By utilizing TRIZ, hospital problems can be examined with a more open view, Go beyond The conceptual framework of the organization and responded more quickly to patients ’ needs.

## 1. Introduction

Quality is so important that is considered as a noticeable concept in our real lift (Mohammad & Alhamadani, 2011) and has become and agenda in management as an effective and pervasive strategy (Sahneya, Banwet & Karunesa, 2006). Quality can be considered as a much important element for making difference in corporate competition environment (Altunaş & Yener, 2012). Quality improvement for service organizations to satisfy service receivers’ expectations and satisfying them has become a challenge nowadays (Prattana, Nattapan, Patchaya, & Kanokporn, 2012). It is believed that improvement of quality of organization’s functionality, is of important approaches in development (Sedighi et al., 2005). Healthcare quality is an important factor in improving patient satisfaction. Also Good quality cares are much vital for reaching Millennium Development Goals (MDGs). Healthcare managers need to have a thorough understanding of the practical enhancement of the cares (Sodani, 2011). Davis et al. (2005) have considered quality measurement as a necessity in competitive environment. Also Lee, Hsu and Chang (2007) suggest that quality of service measurement is first and most important factor in improvement of healthcare quality. Hospitals are organizations Established in order to respond people’s health needs. Paying attention to people’s expectations in order to continue the optimized correlation between suppliers and demanders of services will result in hospital activities’ quality improvement (Majid Pour & Naraghi, 2002). Patient satisfaction is increasingly considered as one of the important factors in measuring healthcare quality (Kurpas & Steciwko, 2005). Satisfaction is customer’s respond to fulfilling his needs. In fact, the level of customer satisfaction with regards to consumption of goods and services that satisfies his needs indicates his satisfaction (Andaleeb & Conway, 2006). Thorough analyzing the differences, managers can clearly determine when and in which dimension of service, recipients’ expectations and experiences aren’t compatible with each other and may lead to dissatisfaction (Karydis, Komboli, & Pannis, 2001).

TRIZ is an innovation and SERVQUAL is a structured quality methodology. Using them together inventive problem solving tools can be used to improve the quality and the highest degree of quality can be achieved with the inventive quality improvement methodology. SERVQUAL instrument is widely used to measure the quality of service systems (Babakus & Mangold, 1992; Vandamme & Leunis, 1993; Lam, 1997; Lee, 2005; Çaha, 2007; Zaim et al., 2010).

Parasuraman, Zeithaml and Berry (1988) founded the SERVQUAL model based on information collected from 12 concentrated groups of consumers, their expectations (Services expecting to receive) and perceptions (Services actually received) and compared them in 10 dimensions. These 10 dimensions include: tangibles, reliability, responsiveness, communication, credibility, security, competency, understanding costumers, courtesy, and accessibility. These scholars decreased these 10 dimensions to 5 dimensions in their later researches. At the other hand TRIZ has been developed to solve problems related to manufacturing systems. However, a TRIZ model is designed to improve the quality of service (Su & Lin, 2008). Attention and interests to TRIZ is increasing day-to-day (Filippi, Motyl, & Ciappina, 2011). TRIZ is a systematic method for researchers, engineers, staff and decision makers to find creative solutions. TRIZ is an acronym for the Russian expression “Теориярешенияизобретательскихзада” (teoriya resheniya izobretatelskikh zadatch) which in English is rendered as “The theory of inventive problem solving” and occasionally goes by the English acronym TIPS (Altunaş & Yener, 2012).

Regarding the issue of clinical governance, which is now being implemented in many hospitals in the country, clinical audits, is one of the seven topics that are of great importance. Clinical audits are a quality improvement process which in order to improve quality of care and services provided to patients and the results is done to improve and This action is done through the systematic review of the current status and adapting them with explicit standards hence conducting intervention and making changes (Heydarpour, Dastjerdi, Rafii, Sadat, & Mostofian, 2011). In addition, patient’s satisfaction and their opinions about the quality of services in hospital is a valid indicator for measuring the quality of services in hospitals, and also awareness of lack of consent in patients provides good opportunities to improve the quality of services at the hospital. Accordingly, the scholar was motivated to conduct researches in hospital in the field of evaluating patient satisfaction using SERVQUAL service quality gap and provide necessary innovative solutions systematically. In this way, taking the advantage of the SERVQUAL questionnaire, scholar has tried to identify and rank the gap between costumers’ expectations and the actually received services and using TRIZ model to improve the strategies needed to improve quality of the services provided to patients. In fact this study represents what’s the priority in quality dimensions of the care provided to patients. And how various TRIZ tools can be used to increase the quality of services provided to patients and improve them.

## 2. Method

This Study is an applied research which used a dynamic qualitative descriptive survey method during year 2011. Statistical society includes every patient who visited the hospital from March 2011 (Beginning of the research).

In this research Cochran’s formula was used in order to count the statistical samples and sample’s volume was set to 96 choices.

Z=1.96; p=q=0.5; Reliability Factor was equal to 95% and the acceptable error d=0.1.


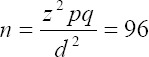


Sampling method is of group (categorized) type and it’s in the simple random groups.

Group sampling is based on patients hospitalized in different units of hospital and their companions at morning, afternoon and night shifts. Existing units include: Emergency; ICU; Urology; Men and Women’s Orthopedics; Men and Women’s Surgery and ICU 1 and 2 of one of Qazvin University of Medical Sciences’ Hospitals in Iran.

In order to gather the information to measure the Quintet dimensions of quality, 7-level Likert SERVQUAL Standard questionnaire was used. 5 dimensions consist of: Tangible factors, Reliability, Responsiveness, Reassurance, and Empathy.

a) Tangible Factors: Existence of Physical facilities, Equipment, Staff and Communication devices.
b) Reliability: The ability to perform the committed service, reliably and accurately.
c) Responsiveness: Willingness to help consumers and provide quick service.
d) Reassurance: Knowledge and courtesy of employees and their ability to build confidence and trust in consumers.
e) Empathy: Attention and Handling of individual customers (Parasuraman et al., 1988).

Validity and Reliability of this questionnaire have been checked and confirmed in different studies in Iran (Kebriaei & Roudbari, 2005). Meanwhile Cronbach Alpha calculation method is used to check the questionnaire’s reliability.

In TRIZ to solve the management issues a 31×31 matrix known as WIN-WIN is used. In this level, it’s enough to select appropriate equivalents for each one of components of the resulting pattern (Variables, Relations, etc.) based on themselves, with attention to its principals and rules in relation with 31 parameters of designing TRIZ.

Based on Ideal plan inappropriate secondary results are located in columns and Parameters which are necessary to be improved are located in rows. In the lanes formed by intersection of rows and columns, two or three principals exist, from the total 40 innovative principals, that can be used to find the innovative solution.

In order to gather information with Presence in the desired units of the hospital, questionnaires were distributed and most of the questions were filled through interview in order to make sure of exact understanding of the questionnaire’s questions by the responder and find the real and true score. Finally all of 96 questionnaires were gathered.

In this research every question and desired variables in the research was studied using descriptive statistical methods of distribution tables etc. According to evaluation of each choice, the possibility to calculate statistical indices regarding to each one the questions is provided.

In this research, scholars have passed the following levels to analyze the information:

a) Calculating mean of each one of the indices of quality, before receiving services
b) Calculating mean of each one of the indices of quality, after receiving real services
c) Calculating the numerical difference between means of each index (as that index’s gap)
d) Sorting gap values calculated for indices, in descending order
e) Assuming the lowest index numerically as the problem, based on the obtained ranking
f) Formulating the desired problem in terms of a contradiction
g) Adaptation of the obtained contradiction with the contradictions matrix’s parameters
h) Establishment of Ideal plan and using the TRIZ contradictions matrix
i) Providing the necessary solutions with reference 40 principals of TRIZ

## 3. Results

56.2 percent of the studied patients (54 persons) are female and 43.8 percent (42 persons) are male. The rest of the characteristics are reported in Table 1.

**Table 1:** Background characteristics of the patients

Percent	Frequency (N =96)	Categories of characteristics	Characteristics studied
Sex	Male	54	56.2
	Female	42	43.8
Age	15-24 YRS	11	11.45
	25-34 YRS	33	34.37
	35-44 YRS	26	27.05
	45-54 YRS	16	16.67
	> 55 YRS	10	10.42
Education Level	Midelscop	48	50
	Diploma	39	40.62
	Bachlor & Over	9	9.38
Job	Free	26	27.08
	Unemployed	14	14.59
	Employee/Worker	38	39.58
	Housekeeper	16	16.67
	Student	2	2.08

Based on Table 2, the Results of quality of the services in view of patients based on SERVQUAL model, shows that in 5 dimensions of quality, the average of perception indices is less than expectations and this shows that patients expect higher quality for the provided services. Negative mark for quality in the results, shows that quality gap exists in every dimension and actually in every item, provided services, couldn’t satisfy patients’ expectations.

**Table 2:** Average and gap of quality of services dimensions based on SERVQUAL model

Measured Dimension	Average of each Dimension after receiving services (Perceptions) P	Average of each Dimension after receiving services (Expectations) E	Gap amount (P-E)	Freedman Test Mean Rank
Tangibles	2.7214	4.6719	-1.9505	3.94
Reliability	3.8000	4.7667	-0.9667	2.88
Assurance	4.5391	5.4297	-0.8906	3.00
Responsiveness	4.8464	5.9896	-1.1432	2.92
Empathy	5.1958	5.7896	-0.5938	2.27

Based on Freedman’s Test which is used for ranking of sequential tests using software systems, highest gap is related to Tangibles and lowest gap is related to Empathy. Therefore the problems which are to be solved using TRIZ method are the problems related to the Tangibles.

The results related to solving the problem using TRIZ, also shows that the 1st step in problem solving using TRIZ method is transforming it to a contradiction. With notice to the fact that in the conducted study tangibles has the highest priority between the several quality dimensions, and considering studied factors in this dimension in SERVQUAL model, the existing contradiction is checked:

Modern and up to date equipment; Appealing and noticeable appearance of physical facilities; Adorned staff with suitable and tidy appearance; Arranged, trim, and clean service providing environment (which are variables of “Tangibles” dimension) are of factors that are costly if created in the best possible manner (weakening factor). Also hospital, According to its mission and in order to survive, must be concerned about their own money making and this won’t happen unless with increase in patients’ demand and feedback (Improvement factor). The hospital either needs to increase its income and decrease its expenses.

With reference to Non-technical contradictions Matrix and synchrony of this two attenuation and improvement factors with it, we will reach to this contradiction:

When supply’s expenses increase, income, demand, and costumer’s feedback decrease.

Next step is to reach to ideal plan.

Table 3 show the TRIZ idealistic plan in the Tangibles dimension. Improvement factor or system’s main function is increase in income, demand and patient’s feedback and attenuation factor is all of the expenses and system’s harmful functions.

**Table 3:** TRIZ idealistic plan in the tangibles dimension

Income, Demand, Costumer Feedback (21)

Supply costs (12)

The next step is to use the Non-technical contradictions Matrix. From encounter of two improvement and attenuation factors, necessary principals for solving the problem are obtained (Rows are improvement factors and columns are attenuation factors). Also each one of quality dimensions can be expressed as a problem in TRIZ template, and obtain TRIZ’s general and proposed solution. Contradictions existing for each one of quality dimensions are shown in Table 4.

**Table 4:** Idealistic TRIZ plan in quintet dimensions of hospital service quality

Service quality dimension	Improvement or attenuation factor	Idealistic plan based on Contradictions Matrix	Principals obtained from idealistic plan
Reliability	Improvement Factor	Income, Demand, Costumer Feedback (21)	37-25-24-3
	Attenuation Factor	Support Expenses (17)	
Tangibles	Improvement Factor	Income, Demand, Costumer Feedback (21)	25-13-35-2
	Attenuation Factor	Supply Expenses (12)	
Responsiveness	Improvement Factor	Income, Demand, Costumer Feedback (21)	25-13-35
	Attenuation Factor	Supply Time (13)	
Assurance	Improvement Factor	Income, Demand, Costumer Feedback (21)	24-12-10-2
	Attenuation Factor	Tension and Pressure (30)	
Empathy	Improvement Factor	Income, Demand, Costumer Feedback (21)	13-40-17-16
	Attenuation Factor	Support Intermediates (20)	

With reference to Business Contradictions Matrix and hospital related experts and professors’ opinion, in all of 5 quality dimensions, the improvement factor is Income/Demand/Costumer Feedback.

Referring to Table 4, if only Tangibles dimension is chosen for improvement, only principals 2-35-25-13 out of 40 principals of TRIZ will be checked and adapted. If we seek an improvement in all of dimensions, all the principles in Table 4 regarding to all aspects of quality can be used. To do so, we summarize the solutions found in Table 4 and after sorting them by frequency and identification of priorities, particular solutions are obtained with reference to the obtained principles and adapting them with the type of hospital business.

With reference to Contradictions Matrix, most important principles between forty-fold principles of TRIZ (Table 5) regarding to Tangibles are principles (2: Taking out); (13: The other way round); (25: Self-service); (35: Parameter change) and the most repeated principle in 5 dimensions of quality in addition to these 4 principles was principle (24: Intermediary). Noticing that in all 5 dimensions examined by SERVQUAL model, we see a negative gap, it’s better to use other principles too, when needed. Finally all of these principles are adapted with hospital-related principles and necessary creative solution was extracted.

**Table 5:** Frequency of forty-fold principles of TRIZ

Principle Name	Principle Number	Frequency
Self-Service	25	3
The other way round	13	3
Intermediary	24	2
Taking out	2	2
Parameter change	35	2
Local quality	3	1
Preliminary action	10	1
Equipotentiality	12	1
Partial or excessive actions	16	1
Another dimensions	17	1
Thermal expansion	37	1
Composite materials	40	1

## 4. Discussion

The quality of service is increasingly important for today’s business, especially in industries with high participation of clients, such as health care and financial services. This can be considered as an essential strategy for reaching to a competitive advantage and increasing long-term profitability (Prattana et al., 2012). In present day’s competitive environment of health care, quality measurement has become a necessity (Davis et al., 2005). Due to tremendously increasing costs, many hospitals are trying adopt quality initiatives to improve their service operations (Prattana et al., 2012).

In Iran, a lot of researches have been conducted in different service organizations using SERVQUAL method. But there aren’t many researches available providing effective and structured solution which can satisfy our need to solving hospital-related problems. On the other hand, regarding the issue of clinical governance, and setting a framework which obligates health service providing organizations to uphold the principles of excellence in clinical services and through this way make them responsible for maintenance and improvement of quality of services they provide (Heydarpour et al., 2011). Providing innovative and systematic solutions can help administrators to improve quality of service to patients.

Present research is looking for studying two main goals including priority of dimensions of quality in view of patients and visitors of studied hospital and then providing innovative solutions.

Based on conducted researches, using SERVQUAL tools, the gap in quality of services provided in the studied hospital is obvious in all 5 dimensions. Highest gap was for Tangibles with an average of 1.9505 and lowest gap was for Empathy with an average of 0.5938. In a research, patients’ expectations are more that nurses in all dimensions, and most expectations are of Tangible dimension. In total result of the study, shows that there is a significant gap between patients’ expectation and nurses’ expectation from nursing services (Azarbayjani, Attafar, Abbasi, & Amirnejad, 2012). Also in another study in Iran, the most gap of quality was in Tangibles (0.68) and the least gap was in Empathy (0.59) and there was a significant difference in perceptions and expectations of visitors (Gholami, Nori, Khojastehpour, & Askari, 2011). While in a research conducted in hispitals of Kashan in Iran, Physician’s Empathy earned the highest expected average and lowest average of quality was earned by tangibility of services (Sabahi-Bidgoli et al., 2011). In a research done in Thailand, results show that generally average of quality of services is positive, yet generally there’s no significant difference between perceptions and expectations of patients’ in the dimension of tangibility (Prattana et al., 2012).

The results of a study in Turkey shows that while tangibility, reliability, courtesy and empathy are important for customer satisfaction, responsiveness and assurance are not (Zaim et al., 2010).

Using the results obtained from Friedman test in this study, it’s obvious that there is a significant gap between patients’ expectation from physical appearance and what they really understood. In other words, the very main dimension of quality which hospital should revise it is hospital’s appearance. But, the notable point is that empathy of staff and patients’ wasn’t relatively much far and it can be noticed as the strength of staff and hospital, although their expectations are not satisfied. Still, we can consider this dimension as the least significant factor in try to improve it in final levels. The results of another study shows that there are some differences between average scores of patients’ perception and expectation from quality of services of medical centers of city of Zahedan, in all dimensions of quality. This means that medical centers couldn’t satisfy patients’ exception in any of fivefold dimensions of quality and perceived quality was always less than expected quality. Results of this study show that there is gap between the present situation and the favorable situation of quality of medical services which can be decreased with planning, management, and proper education (Jenaabadi, Abili, Nastiezaie & Yaghubi, 2011). In a study examining Greek patients’ perceptions and exceptions about quality of dental care, results showed that there is significant gap between patients’ perceptions and exceptions (P = 0.01). The maximum quality gap was in observed in “Responsiveness” dimension. Middle class and lower class women had higher expectations comparing to men of the same class, whereas in higher class, men had higher expectations. This differences were statistically significant (P = 0.02) (Carydis, Komboli-Kodovazeniti, Hatzigeorgiou, & Panis, 2001). Mohammad and Alhamadani (2011) believe that five dimensions of service quality have significant influence on customer satisfaction. These dimensions include tangibles, reliability, responsiveness, assurance, and empathy. Moreover, our findings show that service quality is an important antecedent of customer satisfaction.

In line with next goal, in this research, efficiency of TRIZ in solving non-technical problems through research action in order to create a solution for improving hospital quality issues was tested. The issue was found using SERVQUAL mode, classical TRIZ method was used using the non-technical contradictions in definition of the issue and the idealistic plan as mind navigator in problem solving. As an innovative method, TRIZ can be concomitant and helpful. Azadeh et al., in their study believe that in most organizations, after the establishment of quality control, no significant improvement is observed and this results to a negative attitude towards these standards.

TRIZ creative toolbox of knowledge, attempts to identify and solve the problems using an approach of innovation in organization as one of these tools (Azadeh & Sadeghian, 2010). Chiou, Chien and Tsai (2012) in their study which was done using TRIZ, concluded that innovation methods in provided services can effectively improve the quality of service design. In several researches, TRIZ has been studied along with other methods including QFD etc. (Su & Lin, 2008). Due to goal and results of this study for using SERVQUAL and TRIZ methods in order to improve quality of hospital services, results of Altunaş’s research confirms this new approach (Altunaş & Yener, 2012).

## 5. Conclusion

In this study, quintet dimensions of quality including Reliability, Assurance, Responsiveness, Tangibles, and Empathy were studied. As the results show there was negative gap in all 5 dimensions. Using the obtained data and based on Friedman test, ‘Tangibles’ had the 1st priority in improving patients’ satisfaction. After that, ‘Responsiveness’ had the 2nd priority and ‘Reliability’, ‘Assurance’, and ‘Empathy’ respectively had the next priorities in patient satisfaction. TRIZ creative solutions including contradiction matrix, idealistic plan and principles(13: The other way round); (25: Self-service); (35: Parameter change); (2: Taking out) and (24: Intermediary) out of forty-fold principles of TRIZ were helpful and practical results were achieved from this template. Also a method to use TRIZ in these kinds of problem was provided. Results of this study led to two important points. First, ability of TRIZ to improve hospital service quality; this ability helps administrators of health and treatment organizations specially officials of hospitals and academic centers in decision making. Second, necessity of necessity of using new methods in evaluation of hospitals’ service quality which probably will have better results comparing to traditional models derived from gap analysis model. Using TRIZ hospital’s problems can be analyzed with a more open view and from another perspective, go beyond the organizational conceptual framework and also use the experiences and solutions, developed in other work and scientific fields and areas and respond patients’ needs, more quickly.

Some solutions based on Tangibles which is chosen as the main problem are regulated and offered below, in terms of regulations in the expert panel, based on conditions of the studied hospital for each related principal.

## 6. Recommendations

**Table 6:** Tangible solutions based on adaptation of TRIZ principals for studied hospital

TRIZ Principal	Tangibles’ Solution
Principal 2: Taking out	a)Provide good hospital services
	b)Using closed circuit cameras or using glass instead of wall in CPR room for training the students
	c)Quick transfer of critically ill patients to ICU
	d)Placing noisy instruments (Air conditioning compressors, etc.) outside of building
	e)Setting quiet meeting places in special places
	f)Teleworking and rotation of personnel to prevent their mental depreciation
	g)Outsourcing extraneous hospital activities to other institutions and companies
	h)Quarantine of certain patients in home or in a specific place and separate from people or other patients
	i)Cleaning units’ air using air conditioning devices in hospital
	j)Inviting physicians of good capability and fame, to improve treatment quality in hospital

Principal 13: The other way round	k)Instead of transferring patients outside of center for advice, asking physicians to come to center for advice
	l)Existence of a bank in the hospital
	m)Using radiology and portable laboratory in the emergency unit
	n)Setting up patient guidance system from reception to release

Principal 25: Self-service	o)Using stretcher or chargeable beds
	p)Using personnel in “On Call” mode during crowded shifts
	q)Providing self-services to employees and visitors in hospital’s cafeteria
	r)Installing an ATM in the bank
	s)Installing a snack machine or automatic coin machine in hospital for selling drinks and foods to visitors
	t)Installing automatics doors, which open and close automatically, in hospital entrance and hallways
	u)Installing automatic laser water taps in restrooms
	v)Using automatic door opening and closing systems in hospital’s elevators

Principal 35: Parameter change	w)Changing the flexibility of reception and release in order to minimize the patients’ delay
	x)Provide patients the right to choose their physician
	y)Engage the personnel in strategic plans
	z)Using buzzer or recognized lights in CPR instead of paging and using telephone
	aa)Using forced vacations instead of compressed shifts for personnel to increase their working spirit
